# Knowledge, Utilisation, and Challenges of Medical Doctors Using Picture Archiving and Communication Systems at a Tertiary Academic Hospital in the Eastern Cape, South Africa

**DOI:** 10.1007/s10278-025-01526-2

**Published:** 2025-05-16

**Authors:** Mpatisi Lobi, Anne Faith Namugenyi, Oladele Vincent Adeniyi

**Affiliations:** 1 Department of Radiology, Nelson Mandela Academic Hospital, Mthatha, South Africa; 2 Department of Family Medicine, Cecilia Makiwane Hospital, Mdantsane, East London South Africa; 3 Faculty of Health Sciences, Walter Sisulu University, Mthatha, South Africa

**Keywords:** Challenges with use of PACS, Knowledge of PACS, Picture archiving and communication system, South Africa, Use of PACS

## Abstract

Despite the investments in the picture archiving and communication system (PACS) in the South African health facilities, it is unclear whether clinicians in the rural tertiary hospitals are using this tool maximally. This study determines the level of knowledge, utilisation, and challenges associated with the use of PACS among doctors in a rural tertiary hospital in the Eastern Cape Province. In this cross-sectional descriptive study, a total of 66 medical doctors drawn from different departments completed a structured questionnaire at the Nelson Mandela Academic Hospital, Mthatha. Relevant items on knowledge and use of PACS, including challenges experienced, were obtained. The mean age of the respondents was 36 (± standard deviation 9.58) years. The majority of the doctors (*n* = 42; 63.7%) demonstrated moderate to good knowledge of PACS. Similarly, a substantial majority (*n* = 55; 83.3%) have used PACS for years, for both images and reports (49.2%). The highest proportion of the respondents had at least 1 year of PACS experience (63.5%). Though there was no association between the sociodemographics and level of knowledge, the duration of use (*p* = 0.025) and frequency of use (*p* = 0.025) were significantly associated with moderate to good knowledge of PACS. Internet connectivity and mobile PACS were the major challenges identified. The study found moderate to good knowledge of PACS among the final sample of 66 clinicians. A substantial majority of the clinicians had used PACS for years; however, there is considerable room for strengthening and expanding the use of PACS in the study setting.

**Supplementary Information:**

The online version contains supplementary material available at 10.1007/s10278-025-01526-2.

## Introduction

The picture archiving and communication system (PACS) is a technological advancement that has revolutionised healthcare systems globally. Radiology and telemedicine are the dominant users of PACS for digitisation, archiving, and use of imaging modalities [1–3]. Images are stored as DICOM (Digital Imaging and Communications in Medicine), a universal file format for all vendors developed by the National Electrical Manufacturers Association (NEMA) and the American College of Radiology (ACR). It allows images to be shared and used across various types of networks and PACS vendors [4, 5].

Combined with the radiology information system (RIS), electronic medical records (EMR), and the hospital information system (HIS), PACS constitutes a filmless and paperless healthcare record system [5–7]. Through local connections, internet connections, local servers, cloud storage, and workstations, PACS allows access to imaging modalities and radiology reports from various points of care, such as in-patient wards, outpatient departments, intensive and critical care units, and emergency units in hospitals [2]. This process has improved workflow, patient care, and costs and has also provided a useful education platform for healthcare practitioners [3, 8–10]. Loss of images has been reduced by the use of PACS, as it uses redundancy strategies such as storage of data on site servers for local intranet in real-time access as well as back-up storage in a cloud space. Cloud servers allow access to PACS through computers, cell phones, and tablets [5]. A Steve Biko Academic Hospital (SBAH) study on PACS as an education adjunct for clinicians and medical students demonstrated more imaging educational opportunities in SBAH than in institutions that did not have PACS [3]. There are direct and indirect costs of PACS. Many studies include different components in their cost analysis, such as personnel, equipment, purchase price of PACS, maintenance, and storage space. PACS has reduced costs by eliminating the need for film imaging and thus storage rooms, chemicals, and labour [11, 12]. Filmless radiology has considerably reduced imaging costs; however, the capital outlay for PACS installation is high, as shown in a Durban study comparing the performance of two private hospitals: one with PACS and the other with conventional radiology, which demonstrated reduced total imaging cost in the hospital with PACS [13].

Despite the documented benefits, several factors impact negatively on the optimal use of PACS in different settings. These factors may be categorised as organisational (accessibility, integration with other systems and maintenance), individual (computer literacy, demographics, employment level/position, staff training and resistance), or technological (ease of use and technical problems), with its efficacy also affected by awareness of its benefits, or the lack of it, on the part of clinicians [14–18]. Given that training directly informs knowledge, Saidu et al. [19] demonstrated a clear association between awareness of its benefits and the use of PACS in a study conducted in Nigeria, where trained participants showed more acceptance and willingness to use it than those who had not benefited from such training.

South Africa has a multilevel public healthcare system, from primary healthcare clinics to district hospitals, regional hospitals, and tertiary hospitals. The government and private healthcare companies have invested in the use of PACS in South African healthcare facilities over the past years, and the system is readily accessible to clinicians in many regional and tertiary centres [20]. In spite of the operation, PACS still lags behind compared to western countries. Various post-implementation studies on PACS demonstrated poor knowledge of information technology, lack of training, and computer illiteracy among other barriers [3, 21, 22] In particular, the Eastern Cape Provincial Department of Health installed PACS in most tertiary hospitals in 2015. This was integrated with RIS and made available to radiologists and clinicians at their workstations at various points of care, with web-based PACS available for remote access. PACS is also used in combination with telemedicine for patient discussion with lower-level hospitals [21]. However, clinicians frequently visit the radiology department to access images and print reports, raising concerns about their awareness, knowledge, and use of PACS at their points of care. To date, there has been no study evaluating barriers to the use of PACS in healthcare service delivery in the province. A study was conducted that assessed the use of information and communications technology (ICT) applications for eHealth solutions in rural healthcare, involving five Eastern Cape hospitals [18]. The study found that both structural and psychological factors affected the use of ICT in these centres. The current study determined the level of knowledge, use, and challenges associated with PACS among doctors in a rural tertiary hospital in the Eastern Cape Province. Findings from this study could identify areas that require strengthening for PACS to be used optimally in tertiary hospitals in the province. The findings could also guide strategies for a proposed expansion of PACS to district hospitals and community health centres in the region.

## Methodology

### Study Design and Setting

We conducted a cross-sectional survey of medical doctors across the various clinical disciplines in the Nelson Mandela Academic Hospital in Mthatha. This is a training institute affiliated with Walter Sisulu University for undergraduate medical students and registrars, as well as for research and community engagement. NMAH serves as a referral centre for a population of about three million people residing in the eastern regions of the Eastern Cape Province, comprising OR Tambo Municipality and Alfred Nzo Municipalities. It has a bed count of approximately 736 beds and provides a range of specialist and sub-specialist services. Its many departments include a 24-h accident and emergency unit, an outpatient department, operating theatres, an intensive care unit for adults, paediatrics and neonates, high-care wards, and departments for general surgery, internal medicine, obstetrics and gynaecology, paediatrics, anaesthesia, orthopaedics, ophthalmology, ENT, dermatology, urology, neurology, neurosurgery, oncology, cardiology, and vascular surgery.

### Study Population

The hospital has a robust workforce of 273 medical doctors, 75 of whom were specialists, with the rest being medical officers and registrars during the period of the survey. About eighty intern doctors worked at the hospital during the study period. Note that medical doctors in the Department of Radiology were excluded from the study.

### Sample Size and Technique

A sample size of 204 was calculated, using Cochran’s formula for cross-sectional studies:

$$N=Z1-\propto/2p\left(1-p\right)/d^2$$

(where *z*1 − * ∝ */2 is the standard normal variance at 1.96, ∝  = 0.05; *p* = 0.5).

This sample size was calculated based on the total estimate of doctors across the various departments (or ‘clusters’) (*N* = 355) at a confidence level of 95%, accepting a margin of error of ± 5% and a population proportion of 50%. In anticipation of missing or incomplete responses to the key outcome measures, the sample size was increased by 10%, bringing the total sample size to 204. A multi-stage cluster sampling technique was applied. Self-administered questionnaires, adapted from Tshalibe et al. (2021) and from the literature, were distributed to the heads of department, along with a request letter and consent forms. The questionnaires were distributed proportionally to the size of each cluster and conveniently distributed within the cluster.

### Data Collection

A total of 204 questionnaires were distributed proportionally to each department or cluster, depending on its size, from 15 July 2024 to 6 September 2024. Of the 204 questionnaires distributed, a total of 66 complete responses were included in the data analysis, giving an overall response rate of 32%. The questionnaire comprised four sections: sociodemographic information, level of knowledge, use of PACS, and challenges and recommendations (made up of open-ended questions). The questionnaire had been validated in two previous studies that had Cronbach’s alpha results of 0.7 and 0.98, respectively [17, 20]. We also piloted the questionnaire in the study setting with 10 medical doctors conveniently sampled, which gave a calculated Cronbach alpha score of 0.98. The piloted samples were not included in the main study.

## Measures

### Main Outcomes

The levels of doctors’ knowledge and their use of PACS were assessed in two separate sections, with ten questions for assessment of knowledge. Responses were given on a 5-point Likert scale, ranging from ‘strongly agree’ to ‘strongly disagree’. This covered basic functions to advanced manipulation of imaging and search functions.

Use of PACS Questions in this section also covered the duration of use so far, ranging from weeks, months, to years, along with the frequency of use, ranging from daily, weekly, to monthly. Reasons for the use of PACS were assessed by participants choosing from three possible options: reports, images, or both. Those who had no access to PACS ticked the category ‘no usage’. Another component for the assessment of use was the preferred device, with options given as hospital personal computer, laptop, or mobile device, and the preferred location, with options given as computers in their department, the radiology department only, or in both departments equally.

The fourth component of the questionnaire invited participants to express the challenges they experienced in using PAC and their recommendations on how to improve its use in the various departments. Qualitative responses from the pilot test were coded and categorised into themes. These themes were converted to respondent options (quantitative data) in the questionnaire for the main study.

### Covariates

The questionnaire began with questions on sociodemographic aspects, including age, gender, position, and experience in the use of PACS for patient care. Age of participants in years was categorised as ≤ 36 years, 37–46 years, and ≥ 47 years old. Professional position or category included intern doctor, medical officer or registrar, and consultant. Experience in the use of PACS was categorised in years as no experience, below 1, 1–4, and 5 and above.

### Statistical Analysis

The collected data was entered into an excel spreadsheet, cross-checked and cleaned, then exported to the IBM Statistical Package for Social Sciences Version 29.0 for Windows (IBM Corp., Armonk, NY, USA). Frequencies and percentages were reported for categorical data, while the mean and median interquartile rang e was reported for continuous variables. Likert scale responses were collapsed into two groups: ‘strongly agree’ and ‘agree’ were reduced to ‘agree’; and ‘strongly disagree’ and ‘disagree’ were reduced to ‘disagree’. ‘Agree’ was then replaced by ‘yes’ as the answer to the question, indicating a high level of knowledge, and a score of 1 was given for the question. ‘Disagree’ was replaced by ‘no’, indicating no knowledge, and was given a score of 0 for the question. Each participant was given a point for each ‘yes’ answer in the knowledge domain. An aggregate score for knowledge was determined and classified as: no knowledge (0 correct answers), poor knowledge (< 5 correct answers), moderate knowledge (5–7 correct answers) and good knowledge (> 8 correct answers) (Suppl. 1). The relationship between knowledge and use, as well as the relationship between knowledge and sociodemographic variables, was evaluated with the Fisher exact test. A *p*-value < 0.05 was considered statistically significant.

## Results

### Sociodemographic Characteristics of the Participants

Table 1 presents the sociodemographic characteristics of the participants. More females participated in the study (59%) than males. One participant did not indicate gender. The majority of the participants were in the age group of less than 36 years (59.4%), having a mean age of 36 years, a median age of 34.5 years (± SD 9.58), a minimum age of 24, and a maximum age of 60. All departments participated in the study; however, the highest number of participants came from paediatrics and general surgery, at 15.4% and 10.8%, respectively. Two participants did not disclose their department. The medical officer and registrar category accounted for the majority of the respondents (61%) while consultants were in the minority (12.3%). The highest proportion of the respondents had at least 1 year of PACS experience (63.5%); 38.1% had 1–4 years’ experience and 25.4% had ≥ 5 years’ experience (25.4%) (Table 1). In the weighted adjustment, there was a slight over-sampling (17 instead of 13) among the interns and under-sampling (8 instead of 14) among the consultants (Supp. 1). Similar variations were observed in some departments (Supp. 2).

**Table 1:** Sociodemographic characteristics of participants

Variable	Frequency (*n*)	Percentage (%)
**Age (years)**		
≤ 36	38	59.4
37–46	17	26.6
≥ 47	9	14.1
**Gender**		
Male	26	40
Female	39	59
**Department**		
Internal Medicine^1^	5	7.7
Obstetrics/Gynaecology^2^	5	7.7
Oncology^1^	4	6.2
Paediatrics^1^	10	15.4
Anaesthesia^1^	5	7.7
Dermatology^1^	5	7.7
Accident and Emergency/Staff Clinic^2^	2	3.1
Orthopedic Surgery^2^	2	3.1
ENT^2^	5	7.7
Urology^2^	5	7.7
General Surgery^2^	7	10.8
Neurosurgery^2^	1	1.5
Ophthalmology^2^	4	6.2
Neurology^1^	1	1.5
Psychiatry^1^	4	6.2
**Position**		
Interns	17	26.2
Medical officer/registrar	40	61.5
Consultant	8	12.3
**PACS experience (years)**		
No experience	4	6.3
< 1	19	30.2
1–4	24	38.1
≥ 5	16	25.4

*PACS*, picture archiving and communication system. ^1^Medical. ^2^Surgical

### Level of Knowledge on PACS Among Doctors in the Study

The majority of respondents had a moderate (*n* = 24; 36.4%) to good aggregate score in the knowledge domain of PACS (*n* = 18; 27.3%). Four participants reported a zero level of knowledge of PACS (6.1%) (Suppl. 3).

For knowledge items, 53 respondents (80.3%) reported an ability to search for patients using PACS. In addition, 76.9% of participants reported an ability to filter a patient search list by date. The majority of the respondents confirmed that they could access images on PACS (78.8%) and zoom in and out of images on PACS (75.8%). Contrastingly, fewer respondents could contrast or window images on PACS (42.4%), compare images side by side (34.4%), access online PACS (47%), access PACS on a cell phone or tablet (42.4%), or knew how to use the RIS (16.9%). A slight majority could retrieve reports from PACS (53%) (Table 2).

**Table 2:** Level of knowledge of PACS among doctors in the study

Variables	Frequency (*n*)		Percentage (%)
Ability to search for a patient on PACS			
Yes	53	80.3	
No	13	19.7	
Ability to filter patient search list by date			
Yes	50	76.9	
No	15	23.1	
Ability to zoom in and out images on PACS			
Yes	50	75.8	
No	16	24.2	
Ability to access images on PACS			
Yes	52	78.8	
No	14	21.2	
Ability to contrast or window images on PACS			
Yes	28	42.4	
No	38	57.6	
Ability to compare images side by side			
Yes	22	34.4	
No	42	65.6	
Ability to retrieve reports from PACS			
Yes	35	53.0	
No	31	47,0	
Ability to access online PACS			
Yes	31	47.0	
No	35	53.0	
Ability to access PACS on cell phone or tablet			
Yes	28	42.4	
No	38	57.6	
Knowledge about RIS			
Yes	11	16.9	
No	54	83.1	

*PACS*, picture archiving and communication system; *RIS*, radiology information system

### Level of Utilisation of PACS Among Doctors in the Study

A substantial majority of respondents had used PACS for years (*n* = 55; 83.3%); only four respondents had never used PACS. About 70% of the participants used PACS daily (59%) or weekly (28.8%). More than two-thirds of the participants used the hospital computer to access PACS (69.8%), followed by mobile phones (22.2%). About two-thirds of the respondents accessed PACS via their departmental and radiology department computers equally (65.2%), with a quarter confirming their use of PACS through their departmental computers only. The main reasons for accessing PACS were to retrieve images (33.8%), results (12.3%), or both (49.2%) (Table 3).

**Table 3:** Level of utilisation of PACS among doctors in the study

Variable	Frequency (*n*)	Percentage (%)
**Duration of PACs usage**		
No usage	4	6.1
Weeks	0	0
Months	7	10.6
Years	55	83.3
**Frequency of PACS usage**		
No usage	4	6.1
Daily	39	59
Weekly	19	28.8
Monthly	4	6.1
**Reason for usage**		
No usage	3	4.6
Reports	8	12.3
Images	22	33.8
Both	32	49.2
**Device used for access**		
Hosp PC	44	69.8
Laptop	2	3.2
Mobile	14	22.2
Hosp PC, mobile	2	3.2
Hosp PC, laptop, mobile	1	1.6
**Preferred location for access**		
No preferred access	1	1.5
Computers in my department only	17	25.8
Radiology department only	5	7.6
Use both departments equally	43	65.2

*PACS*, picture archiving and communication system

Among the medical officers/registrars (*n* = 40), nearly all (*n* = 38; 95%) had used PACS for years. Similarly, six of eight consultants had used PACS for months to years, while all the interns had used PACS for months to years. A substantial majority of the respondents used PACS daily to weekly (87.7%); all the interns used PACS daily to weekly, 87.5% of the medical officers/registrars used PACS daily to weekly, and 62.5% of the consultants used PACS daily to weekly. Half of the consultants and medical officers/registrars used PACS for both images and reports. However, seven of 17 interns used PACS for images only. Hospital computers were used by many of the respondents to access PACS.

In addition, a few medical officers/registrars (11 of 40) used mobile phones to access PACS. Three-quarters of the consultants (75%) and medical officers/registrars (75%) preferred to use both their departmental and radiology department computers to access PACS, while almost three-quarters of the interns preferred to use their departmental computers (47.1%) rather than the radiology department computers (17.6%). The remaining six interns used their departmental and radiology department computers equally (35.3%) (Suppl. 4).

### Correlation of Level of Knowledge with Sociodemographic Characteristics

Participants aged 36 years or below had better knowledge of PACS than participants in other age groups; however, there was no significant association between age categories and the knowledge domain. It was found that males had slightly better knowledge of PACS than their female counterparts, but again, there was no significant difference in the knowledge domain by gender. Similarly, although the surgical departments had slightly better knowledge of PACS than the medical departments, there was no significant difference between them in the knowledge domain. There was also no significant difference between the level of knowledge of PACS among the doctors according to their professional category or their years of experience with PACS (Table 4).

**Table 4:** Correlation between sociodemographic characteristics and level of knowledge

Variable	No knowledge			Poor knowledge			Moderate knowledge		Good knowledge		*p*-value
	*n*	%		*n*		%	*n*	%	*n*	%	
**Age (years)**											0.653
≤ 36	1	2.6		12		31.6	15	39.5	10	26.3	
37–46	2	11.8		5		29.4	5	29.4	5	29.4	
≥ 47	1	11.1		2		22.2	4	44.4	2	22.2	
**Gender**											
Male	2		7.7	5	22.2		8	30.8	11	42.3	0.131
Female	2		5.1	14	35.9		16	41	7	17.9	
**Department**											
Medical	3		8.8	12	35.9		13	38.2	6	17.6	
Surgical	1		3.2	7	22.6		11	35.5	12	38.7	
**Position**											
Interns	0		0	5	29.4		7	41.2	5	29.4	0.423
MO/REG	2		5.0	13	32.5		15	37.5	10	25.0	
Consultants	2		25.0	1	12.5		2	25	3	37.5	
**PACS experience (years)**											
None	2	50.0		1	25.0		1	25	0	0	0.102
< 1 yr	0	0		6	31.6		7	36.8	6	31.6	
1–4	2	8.3		8	33.3		10	41.7	4	16.7	
≥ 5 yr	0	0		4	25.0		4	25	8	50	

*PACS*, picture archiving and communication system; *MO/REG*, medical officer/registrar. Medical = 34 (52%); surgical = 31 (47%)

### Correlation Between Level of Knowledge and Use of PACS

There was a significant association between the duration of use of PACS and the level of knowledge among the doctors. Those who had used PACS for months and years had moderate to good knowledge of PACS (*p* = 0.025). Similarly, there was a significant association between the frequency of use of PACS and the level of knowledge among the doctors. Participants who used PACS daily to weekly had significantly better knowledge than those who used it monthly or not at all (*p* = 0.002). There was no significant association between level of knowledge and reason for accessing PACS, device used for access, and preferred location of access (*p* > 0.05) (Table 5).

**Table 5:** Correlation between level of knowledge and use of PACS

Variables	No knowledge		Poor knowledge					Moderate knowledge					Good knowledge					*p*-value
	(*n*)	(%)	(*n*)				(%)	(*n*)				(%)	(*n*)			(%)		
Duration of PACS^1^ usage																		
No usage	2	50.0	1				25.0	1				25	0			0.0		0.025
Weeks	0	0.0	0				0.0	0				0.0	0			0.0		
Months	1	14.3	1				14.3	2				28.6	3			42.9		
Years	1	1.8	18				32.7	21				38.2	15			27.3		
Frequency of PACS^1^ usage																		
No usage	3	75	0			0.0		1			25.0		0				0.0	0.002
Daily	0	0.0	11			28.2		16			41		12				30.8	
Weekly	0	0.0	7			36.8		7			36.8		5				26.3	
Monthly	1	25	2			50.0		0			0.0		1				25.0	
Reason for usage																		
No usage	2	66.7	0		0.0			1			33.3		0			0.0		0.009
Reports	0	0.0	4		50.0			2			25.0		2			25.0		
Images	1	4.5	7		31.8			12			54.5)		2			9.1		
Both	1	3.1	8		25.0			9			28.1)		14			43.8		
Device used for access																		
Hosp PC^2^	1	2.3	14		31.8			18		40.9			11		25.0			0.121
Laptop	1	50	1		50			0		0.0			0		0.0			
Mobile	0	0.0	3		21.4			4		28.6			7		50.0			
Hosp PC^2^, mobile	0	0.0	2		100			0		0.0			0		0.0			
Hosp PC^2^, laptop, mobile	0	0.0	0		0.0			1		100			0		0.0			
Preferred location for access																		
No preferred access	0	0.0	1	100.0				0	0.0				0	0.0				0.807
Computers in my department only	0	0.0	6	35.3				6	35.3				5	29.4				
Radiology department only	1	20.0	1	20.0				2	40.0				1	20.0				
Use both departments equally	3	7.0	12	27.9				16	37.2				12	27.9				

*PACS*, picture archiving and communication system; *PC*, personal computer

### Challenges of Using PACS

The majority of the respondents (63.6%) reported experiencing connectivity issues. However, the nature of these issues was not indicated, so it was not possible to tell if the problems arose from hardware inadequacies, local network connectivity, or internet connectivity. However, 28.8% of respondents reported hardware issues, workstations being unavailable in the cluster, or workstations not switching on or not being connected to the network as particular challenges. Power failures were also raised as a problem, leading to recurrent connectivity issues. Some departments were off site and therefore had no direct connection to the network. Another major concern was mobile PACS (25.7%) not working on some mobile devices. A few participants (12.1%) asked for training on how to use PACS. Some respondents (10.6%) raised the issue of difficulty in finding patients owing to incorrect patient details being provided, such as incorrect demographic details and/or spelling errors in their names. Some mentioned the inability to access advanced image manipulation and delays in the availability of reports and images in the system (Suppl. 5).

### Recommendations to Improve the Use of PACS

Most participants mentioned improvements in connectivity as their major recommendation, and better access through mobile devices. New workstations and better maintenance were also recommended. A number of participants (29%) suggested increased training on PACS usage. Some (13.6%) recommended upgrades to the system for advanced image manipulation and offsite access. There was a demand to have prompt availability of the reports in the system. Correct uploading of demographic details and names was mentioned. Mistakes in this area may be attributed to human error in recording details in patients’ files.

## Discussion

The South African government and the private sector have invested considerably in PACS over the past decade. The strategic implementation of PACS in South African health facilities is supported by a body of reliable scientific evidence. With PACS, there are direct and indirect reductions in imaging operational costs, as it eliminates the need to print films and reports, among many other benefits [13]. In addition, PACS has become a useful teaching and learning tool for clinicians and medical students [3]. However, despite widespread access to PACS, it is still unclear whether clinicians in the rural tertiary hospitals in South Africa are using this innovative tool optimally in patient care. Context-specific factors influencing the use of this innovative health technology could inform a strategy for strengthening and expanding PACS across the country’s healthcare facilities. The study determined the knowledge of and use of PACS among doctors at a rural tertiary hospital in the Eastern Cape Province. In addition, the study identified the challenges associated with the use of PACS at the point of care in different departments in the hospital.

Almost two-thirds of the clinicians demonstrated moderate to good knowledge of PACS (63.7%). This is not surprising, given that PACS was installed in the hospital a decade ago, giving doctors time to become accustomed to it. The findings corroborate a previous study in the same setting, which showed that 81% of the doctors acknowledged PACS as a useful innovation in patient care [23]. The level of knowledge displayed by the doctors in this study is higher than that of doctors surveyed in a Myanmar study, which showed that only 20% of clinicians had adequate knowledge of information technology [24]. A relatively high proportion of the participants in the current study (36%) had poor to zero knowledge of PACS; these clinicians are an essential part of the healthcare team and cannot be ignored. A previous study highlighted lack of training, low levels of computer literacy, lack of motivation or interest in information and communication technology, and minimal exposure to technology as gaps to be addressed for clinicians with poor knowledge [15]. Computer literacy has been demonstrated to be associated with the intention to use electronic medical records [25]. A Nigerian study found that there was good knowledge of PACS among participants that had undergone training [19]. However, in a Namibian study, only 30% of clinicians had undergone training on PACS [16]. Therefore, targeted training for doctors with zero or poor knowledge of PACS will bridge identified gaps in the study setting.

Some participants’ clusters in the current study had no PACS infrastructure, so that these participants had minimal exposure to PACS. This is a structural barrier to be addressed by the clinical governance of the hospital. All work areas should have computers installed to increase access to PACS for all doctors in the hospital.

In the knowledge domain, over 75% of the respondents reported knowledge of basic PACS use, having the ability to search for patients, filter by date, access images on PACS, and zoom in and out of images. These findings corroborate those of a previous study from Riyadh, Saudi Arabia, where participants stated that PACS had improved clinical decision making because of their ability to zoom in and out, contrast, and brighten images [26]. However, in the current study, there was poor knowledge of the advanced use of PACS and of PACS components like the RIS. In total, only 16% of the respondents had knowledge of RIS. Similar poor knowledge of PACS features (30%) has been reported elsewhere [16].

The majority of the participants had used PACS for years, and 60% used it daily. Almost two-thirds of the respondents (65.2%) used both their departmental and radiology department computers to access PACS. Common reasons for visiting the radiology departments included the benefits of face-to-face patient discussions with radiologists, image evaluation with radiologists, and printing of reports. Some doctors viewed images in the radiology department without the assistance of the radiologist. A substantial majority of the doctors preferred to use hospital computers to access PACS (69.8%), although some (22%) used their mobile phones. This finding aligns with those of previous studies which found no significant association between the use of smart phones and the conventional use of PACS for viewing images. But the authors of these studies cautioned that the smart phone cannot be a replacement for the conventional way of accessing PACS [27, 28]. It is imperative that clinicians embrace this paperless and filmless innovation in the hospital settings [29]. Most studies on the topic assess user satisfaction with PACS, but few examine the way in which the system is used. A study on acceptance of PACS based on the technology acceptance model (TAM) demonstrated that 94% of participants ‘always’ used PACS [30]. TAM is an information systems theory developed by Davis in 1989 to assess technology acceptance and usage. In order to determine the acceptance of new technology, TAM aims to assess the perceived usefulness and ease of use [30, 31].

This study did not find any clear association between the demographic characteristics of the clinicians and their level of knowledge about PACS. A plausible explanation for this finding could be the relatively long period of PACS use by the older participants. A similar finding was reported in a previous study on PACS, which applied the UTAUT (unified theory of acceptance and use of technology) model of technology acceptance assessment [16]. The majority of the respondents in this study were under the age of 36 years and had moderate to good knowledge of PACS. This contrasts with the findings of a previous study on radiographers, which found that there was good knowledge among younger participants [17]. However, there are many factors that affect knowledge and motivation to use technology apart from age — functional value, emotional value, and epistemic value, among others [32]. The UTAUT model, another theory for assessing acceptance of technology, developed by Venkatesh et al. (2003) explored factors affecting the use of technology, including performance expectancy, effort expectancy, social influence, and facilitation condition [16, 25, 33].

The majority of respondents were female, while other studies have shown a higher percentage of males or an equal number of males and females [16, 17, 34]. The surgical clusters demonstrated better knowledge of PACS than the medical clusters. This finding could be explained by the fact that surgeons use images and/or reports from PACS in planning surgeries for their patients. A content analysis study conducted in three Saudi Arabian hospitals showed that 70% of physicians felt that the PACS system improved their efficiency [26]. An examination of professional level in relation to knowledge level in the current study showed that interns had better knowledge of PACS than medical officers and registrars; however, the difference was not statistically significant. It was also found that the experience of using PACS had no significant relationship with knowledge level; however, 50% of participants who had 5 years or more of experience had good knowledge. In contrast, a previous cross-sectional study assessing ICT knowledge and use among radiographers showed poor ICT knowledge, even among participants with many years’ experience [17].

The majority of participants in this study had used PACS for months to years, and demonstrated moderate to good knowledge, which is statistically significant. This finding demonstrates that there is a relationship between knowledge and regular use, which in turn shows the value of training to increase knowledge and use. An Ethiopian study assessing knowledge and use of ICT in healthcare showed that there was poorer ICT use among participants with more experience in healthcare than among those with fewer years’ experience in healthcare [35]. In the current study, those who used the system daily to weekly had moderate to good knowledge. There was no significant correlation between PACS use and professional category. The majority accessed PACS for both reports and images, as in another South African study on clinicians’ perspective on PACS [20]. A South African study assessed private practitioners’ meaningful use of PACS; 17% of the participants reported a positive impact of PACS usage in their ward rounds. Further, more than 60% of the participants reported a positive impact of PACS usage in their consultations by showing images to their patients. In addition, a reduction in the time spent searching for images and reports, and overall efficiency were highlighted [36].

The majority of participants in the present study mentioned connectivity issues and mobile PACS as their greatest challenges, followed by device and workstation issues. Similar findings were made in other studies [19, 20]. Though, some of the respondents cited connectivity issues as a challenge, the internet speed and type of connectivity were not explored in this study. A case-based study involving five Eastern Cape rural hospitals, including NMAH, found that a lack of both ICT equipment and computer skills were barriers to ICT application in the rural healthcare centres [18]. Some respondents in the current study also mention the non-availability of images and reports, including delayed image uploading. An online longitudinal observational study conducted on social media discussions by PACS experts and users identified similar challenges [37]. One user noted the issue of recurrent electricity shutdowns, which was also noted in two other studies [19, 20]. It is clear that even though PACS and the entire radiology department at NMAH have an uninterrupted power supply and a back-up system, the system does not work perfectly.

The respondents stated that training is required to improve their use of PACS in the study setting. This corroborates similar findings in other studies [3, 17–19, 24, 25]. As most of the participants demonstrated knowledge of basic PACS components, targeted training of the advanced components would be beneficial to improve access to reports and the usage of advanced manipulation of images. A combination of both formal and informal training is recommended. Formal training by the PACS administrator for the clinicians across all the departments should be encouraged. This can be delivered via online webinars. Similarly, the radiology department should provide formal orientation to interns and other new doctors joining the institution. This can be followed by quarterly updates and also monitoring of improvement in the PACS usage periodically. Informal training should complement all other trainings. Especially, new clinicians joining the various departments will receive informal training on the use of PACS by the respective departmental staff. The Baltimore study demonstrated that training was conducted by an onsite application specialist, and the hospital had new physicians rotating monthly through the departments [38].

Similarly, in the current institution, new interns are coming in annually, and new medical officers and registrars are being employed. The training would be done per department, as this would allow for more individual and specific training per departmental needs (specialty or subspecialty). Another study applied a cognitive load theory to simplify learning and training of PACS and suggested visual learning more than plain text to reduce mental effort and improve learning. Segmentation and individualisation were applied, where the information would be presented in short intervals and according to the needs of the trainees [39]. In addition, respondents recommended an upgrade to the system, which should include consistent offsite access by doctors. There is a demand to reduce the waiting time for reports uploading on the system. RIS is used for registering patients, scheduling examinations and procedures, reporting, and storage of reports [37]. Given that a substantial majority of the participants had no knowledge of RIS, there is a need for awareness raising and training on RIS, which also allows access to reports. Some reports may remain on RIS, while others automatically transfer from RIS to PACS once completed.

Steps should be taken to boost efficiency and accuracy in patient documentation, and to link PACS to all hospital information and electronic medical records. Another suggested solution to the problem of inaccessibility at workstations was to improve mobile PACS and web-based PACS for laptops and tablets [20, 37]. The use of identity (ID) numbers could also improve the ease of searching for patients on PACS, as mentioned by a respondent in another study who used patients’ IDs to save time [26]. Another study suggested the creation of a unique identifier, as some patients may not have IDs, and some have similar names [18]. One respondent suggested that Wi-Fi be made available in every part of the hospital for clinicians, which would be beneficial if all devices had access. Improvements in the technical support provided were also suggested, as in other studies [19, 20]. PACS is characterised by a high-speed network with cache-less features to allow immediate access to images and the sharing of images. Its management and maintenance have moved from the radiology department to technology services and clinical governance. Integration with artificial intelligence application is under consideration [40]. An American society of radiology survey among radiologists integrating artificial intelligence (AI) revealed that 94% of the participants said it was inconsistent, while in a European study only 13% said they would acquire it for their work. AI software has to be continuously adjusted and undergo learning [41].

PACS was previously used separately with electronic medical records; however, it has been demonstrated that integration with EMR has shown a reduction in the time radiologists use to access patients’ records, with resultant improved work flow as the records open simultaneously with images. Access to adequate patient information improves diagnostic accuracy [42].

### Strength and Limitations of the Study

This is the first study in the Eastern Cape Province to use a reliable tool with a good Cronbach alpha score to assess PACS’ use among clinicians. The study provides insights on the training needs of doctors in the study setting and has offered some useful recommendations that, if adopted, could greatly enhance PACS usage in the study setting. However, the limitations of the study cannot be ignored. The low response rate among doctors and the cross-sectional nature of the study are notable limitations. Future studies should recruit a representative sample from the clinical departments and across professional categories, especially the consultants. Nonetheless, all the clinical specialties were represented in the study. The speed of internet connectivity was also not assessed, which could have given additional insight on why the doctors preferred the radiology department for viewing images.

## Conclusions

Overall, the study found moderate to good knowledge of PACS among the final sample of 66 clinicians. A substantial majority of the clinicians had used PACS for several years. However, the results show that there is considerable room for strengthening and expanding the use of PACS across the various clinical disciplines in order to relieve the burden on the radiology department. A need for training was noted in order to promote doctors’ use of departmental computers in addition to radiology department computers to access reports and images. Departmental use would not necessarily replace face-to-face patient discussions and imaging reviews with radiologists, as such discussions can enhance understanding of images. The integration of PACS with RIS and the broader hospital information system and electronic medical records would improve users’ experience of PACS. Future studies should monitor longitudinal trends in the use of PACS in the various clinical disciplines. Further studies are suggested to assess the intention to use the latest innovations, including quantum computing, artificial intelligence, and cloud computing, and their application in the radiology community of Eastern Cape province and South Africa.

## Supplementary Information

Below is the link to the electronic supplementary material.

ESM 1 (DOCX. 60.3 KB)

## Data Availability

Data and instruments are available on request from the corresponding author.

## Declarations

### Disclaimer

The views contained in this article are that of the authors and not of the institutions they are affiliated with.
