# Supplementary material for: Knowledge, Utilisation, and Challenges of Medical Doctors Using Picture Archiving and Communication Systems at a Tertiary Academic Hospital in the Eastern Cape, South Africa
Source: J Imaging Inform Med. 2025 May 16;39(1):34–45. doi: 10.1007/s10278-025-01526-2 (PMC12920820; doi:10.1007/s10278-025-01526-2)
Supplement: Supplementary file 1 — (DOCX. 60.3 KB) [file 10278_2025_1526_MOESM1_ESM.docx]

**Supplementary results**

**Supp. 1. Weighted average per professional category**

| **Variables** | **Interns** | **Medical officers/registrars** | **Consultants** |
| --- | --- | --- | --- |
| Desired number (N=66) | 0.191 X 66 =13 | 0.594 X 66 = 39 | 0.211 X 66 = 14 |
| Actual number | 17 | 40 | 8 |
| Weighing factors (WF) | 0.7415294118 | 0.9801 | 1.74075 |

Supp. 2. Distribution of participants in comparison to the proportions expected per department

|  |  | **Estimated total population** | | **Expected sample per department**  **N=66** | **Response** | |
| --- | --- | --- | --- | --- | --- | --- |
| **Clusters** | **Doctors** | **n** | **%** | **N’** | **n** | **%** |
| Anaesthesia | | 45 | 12.7 | 8 | 5 | 7.7 |
| Accident and Emergency | | 9 | 2.5 | 2 | 2 | 3.1 |
| Internal Medicine | | 40 | 11.2 | 7 | 5 | 7.7 |
| ENT/Maxillofacial | | 13 | 3.7 | 2 | 5 | 7.7 |
| Obstetrics/gynaecology | | 48 | 13.5 | 9 | 5 | 7.7 |
| Paediatrics/Neonates | | 52 | 14.6 | 10 | 10 | 10 |
| Paediatric surgery | | 7 | 2 | 1 | 0 | 0 |
| General surgery | | 48 | 13.5 | 9 | 7 | 10.8 |
| Orthopaedic surgery | | 29 | 8.1 | 5 | 2 | 3.1 |
| Plastic Surgery | | 5 | 1.4 | 0 | 0 | 0 |
| Neurosurgery | | 11 | 3.1 | 2 | 1 | 1.5 |
| Ophthalmology | | 10 | 2.8 | 2 | 4 | 6.2 |
| Urology | | 12 | 3.3 | 2 | 5 | 7.7 |
| Oncology | | 6 | 1.7 | 1 | 4 | 6.2 |
| Psychiatry | | 10 | 2.8 | 2 | 4 | 6.2 |
| Dermatology | | 10 | 2.8 | 2 | 5 | 7.7 |
| **Total population/%/Total sample size(n)** | | 355 | 100 | 66 | 66 | 100 |

**Suppl. 3: Aggregate level of knowledge of PACS among doctors in the study**

**Suppl. 4: Utilization of PACS by professional category**

| **Variable** | **Interns** | | **MO/REG^2^** | | **Consultants** | |
| --- | --- | --- | --- | --- | --- | --- |
| **Duration of PACS^1^ usage** | (n) | (%) | (n) | (%) | (n) | (%) |
| No usage | 0 | 0.0 | 2 | 50.0 | 2 | 50 |
| Weeks | 0 | 0.0 | 0 | 0.0 | 0 | 0.0 |
| Months | 5 | 71.4 | 0 | 0.0 | 2 | 28.6 |
| Years | 12 | 22.2 | 38 | 70.4 | 4 | 7.4 |
| **Frequency of PACS usage** |  | |  | |  | |
| No usage | 0 | 0.0 | 1 | 25.0 | 3 | 75.0 |
| Daily | 11 | 28.9 | 24 | 63.2 | 3 | 7.9 |
| Weekly | 6 | 31.6 | 11 | 57.9 | 2 | 10.5 |
| Monthly | 0 | 0.0) | 4 | 100.0 | 0 | 0.0 |
| **Reason for usage** |  | |  | |  | |
| No usage | 0 | 0.0 | 1 | 33.3 | 2 | 66.7 |
| Reports | 4 | 50.0 | 4 | 50.0 | 0 | 0.0 |
| Images | 7 | 31.8 | 13 | 59.1 | 2 | 9.1 |
| Both | 6 | 19.4 | 21 | 67.7 | 4 | 12.9 |
| **Device used for access** |  | |  | |  | |
| Hosp PC^3^ | 15 | 34.1 | 25 | 56.8 | 4 | 9.1 |
| Laptop | 1 | 50.0 | 1 | 50.0 | 0 | 0.0 |
| Mobile | 1 | 7.1 | 11 | 78.6 | 2 | 14.3 |
| Hosp PC, Mobile | 0 | 0.0 | 1 | 100 | 0 | 0.0 |
| Hosp PC, Laptop, Mobile | 0 | 0.0 | 1 | 100 | 0 | 0.0 |
| **Preferred location for access** |  | |  | |  | |
| No preferred access | 0 | 0.0 | 0 | 0.0 | 1 | 100 |
| Computers in my department only | 8 | 47.1 | 8 | 47.1 | 1 | 5.9 |
| Radiology department only | 3 | 60.0 | 2 | 40.0 | 0 | 0.0 |
| Use both departments equally | 6 | 14.3 | 30 | 71.4 | 6 | 14.3 |

PACS = Picture Archiving and Communication System; MO/REG = medical officer/registrar; PC = personal computer

**Suppl. 5. Challenges in the use of PACS**

| **Variables** | **Frequency**  **(n)** | **Percentage**  **(%)** |
| --- | --- | --- |
| Connectivity issues | 42 | 63.6 |
| Device issues/workstations | 19 | 28.8 |
| Mobile PACS issues | 17 | 25.7 |
| Unavailable reports on system | 11 | 16.7 |
| Unavailable image on system | 9 | 13.6 |
| Training issues | 8 | 12.1 |
| Typing errors in patients’ names | 7 | 10.6 |
| No challenges | 7 | 10.6 |
| Advanced imaging manipulation | 5 | 7.6 |
| Unit/departments out of site | 4 | 6.1 |
| Logins differ | 1 | 1.5 |
| System goes off during loadshedding | 1 | 1.5 |
